# Supplementary material for: Heat adaptation of phage T7 under an extended genetic code
Source: Virus Evol. 2021 Dec 1;7(2):veab100. doi: 10.1093/ve/veab100 (PMC8923235; doi:10.1093/ve/veab100)
Supplement: veab100_Supp [file veab100_supp.zip › Sequencing_Analysis.zip!/Sequencing_Analysis/S2/evidence/RA_35.html]

BRESEQ :: Evidence


| Read alignment evidence... | | | | | | | | | | | |
| --- | --- | --- | --- | --- | --- | --- | --- | --- | --- | --- | --- |
|  | seq id | position | | ref | new | freq | score (cons/poly) | reads | annotation | genes | product |
| \* | NC\_001604.1 | 35,515 | 0 | A | C | 48.1% | 1287.0 / inf | 5686 | intergenic (–/–) | *–/–* | –/– |
| Reads supporting (aligned to +/- strand):  **ref** base A (1464/1476);  **new** base C (1385/1345);  **total** (2858/2828) | | | | | | | | | | | |
| Fisher's exact test for biased strand distribution *p*-value = 4.90e-01 | | | | | | | | | | | |
| Kolmogorov-Smirnov test that lower quality scores support variant *p*-value = 8.66e-01 | | | | | | | | | | | |

Only 100 of 5713 total aligned reads displayed.  

`CAGTGGCGCGATGAGACCAAGGGTTTCCGAGACGAAGCCAAGCGGTTCAAGAATACGGCTGGTCAATACGCTACATCTGCTGGGAACTCTGCTTCCGCTGCGCATCAATCTGAGGTAAACGCTGAGAACTCTGCCACAGCATCCGCTAACTCTGCTCATTTGGCAGAACAGCAAGCAGACCGTGCGGAACGTGAGGCAGACAAGCTGGAAAATTACAATGGATTGGCTGGTGCAATTGATAAGGTAGATGGAACCAATGTGTACTGGAAAGGAAATATTCACGCTAACGGGCGCCTTTACATGACCACAAACGGTTTTGACTGTGGCCAGTATCAACAGTTCTTTGGTGGTGTCACTAATCGTTACTCTGTCATGGAGTGGGGAGATGAGAACGGATGGCTGATGTATGTTCAACGTAGAGAGTGGACAACAGCGATAGGCGGTAACATCCAGTTAGTAGTAAACGGACAGATCATCACCCAAGGTGGAGCCATGACCGGTCAGCTAAAATTGCAGAATGGGCATGTTCTTCAATTAGAGTCCGCATCCGACAAGGCGCACTATATTCTATCTAAAGATGGTAACAGGAATAACTGGTACA  >  NC_001604.1/35215‑35815`  

                                                                                                                                                                                                                                                                                                            `|`                                                                                                                                                                                                                                                                                                              
    `ggCGCGATGAGACCAAGGGTTTCCGAGACGAAGCCAAGCGGTTCAAGAATACGGCTGGTCAATACGCTACATCTGCTGGGAACTCTGCTTCCGCTGCGCATCAATCTGAGGTAAACGCTGAGAACTCTGCCACAGCAGCCGCTAACTCTGCTCATTTGGCAGAACAGCAAGCAGACCGTGCGGAACGTGAGGCAGACAAGCTGGAAAATTACAATGGATTGGCTGGTGCAATTGATAAGGTAGATGGAACCAATGTGTACTGGAAAGGAAATATTCACGCTAACGTGCGCCTTTACATGAc                                                                                                                                                                                                                                                                                                          <  1:126730/301‑1 (MQ=255)`  
          `aTGATACCAAGGGTTTCCGAGACGAAGCCGAGCGGTTCAAGAATACGGCTGGTCAATACGCTACATCTGCTGGGAACGCTGCTTCCGCTGCGCATCAATCTGAGGTAAACGCTGAGAACTCTGCCACAGCATCCGCTAACTCTGCTCATTTGGCAGAACAGCAAGCAGACCGTGCGGAACGTGAGGCAGACAAGCTGGAAAATTACAATGGATTGGCTGGTGCAATTGATAAGGTAGATGGAACCAATGTGTACTGGAAAGGAAATATTCACGCTAACGGGCGCCTTTACCTGACCACaaa                                                                                                                                                                                                                                                                                                    <  1:409492/301‑1 (MQ=255)`  
           `tGAGACCAAGGGTTTCCGAGACGAAGCCGAGCGGTTCAAGAATACGGCTGGTCAATACGCTACATCTGCTGGTAACTCTGCTTCCGCTGCGCATCAATCTGAGGTAAACGCTGAGAACTCTGCCACAGCATCCGCTAACTCTGCTCATTTGGCAGAACCGCACGCAGACCGTGCGGAACGTGAGGCAGACAAGCTGGAAAATTACAATGGATTGGCTGGTGCAATTGATAAGGTAGATGGAACCAATGTGTACTGGAAAGGAAATATTCACGCTAACGGGCTCCTTTACATGACCACAAAc                                                                                                                                                                                                                                                                                                   <  1:696830/301‑1 (MQ=255)`  
             `agaCCAAGGGTTTCCGAGACGAAGCCAAGCGGTTCAAGAATACGGCTGGTCAATACGCTACATCTGCTGGGAACTCTGCTTCCGCTGCGCATCAATCTGAGGTAAACGCTGAGAACTCTGCCACAGCATCCGCTAACTATGCTCATTTGGCAGAACAGCAAGCAGACCGTGCGGAACGTGAGGCAGACAAGCTGGAAAATTACAATGGATTGGCTGGTGCAATTGATAAGGTAGATGGAACCAATGTGTACTGGAAAGGAAATATTCACGCTAACGGGCGCCTTTACCTGACCACAAActg                                                                                                                                                                                                                                                                                                 >  1:823022/1‑299 (MQ=255)`  
                    `gggTTTCCGAGACGAAGCCAAGCGGTTCAAGAATATGGCTGGTCAATACGCTACATCTGCTGGGAACTCTGCTTCCGCTGCGCATCAATCTGAGGTAAACGCTGAGAACTCTGCCACAGCATCAGCTAACTATGCTCATTTGGCAGAACAGCAAGCAGACCGTGCGGAACGTGAGGCAGACAAGCTGGAAAATTACAATGGATTGGCTGGTGCAATTGATAAGGTAGATGGAACCAATGTGTACTGGAAAGGAAATATTCACGCTAACGGGCGCCTTTACATTACCACAAACGGTTTTGAc                                                                                                                                                                                                                                                                                          >  1:190829/1‑301 (MQ=255)`  
                    `gggTTTCCGAGACGAAGCCAAGCGGTTCAAGAATATGGCTGGTCAATACGCTACATCTGCTGGGAACTCTGCTTCCGCTGCGCATCAATCTGAGGTAAACGCTGAGAACTCTGCCACAGCAGCCGCTAACTCTGCTCATTTGGCAGAACAGCAAGCAGACCGTGCGGAACGTTAGGCAGACAAGCTGGAAAATTACAATGGATTGGCTGGTGCAATTGATAAGGTAGATGGAACCAATGTGTACTGGAAAGGAAATATTCACGCTAACGGGCGCCTTTACATGACcaactgtatcttatac                                                                                                                                                                                                                                                                                          >  1:197534/1‑287 (MQ=255)`  
                    `gggTTTCCGAGACGAAGCCAAGCGGTTCAAGAATACGGCTGGTCAATACGCTACATCTGCTGGGAACTCTGCTTCCGCTGCGCATCAATCTGAGGTAAACGCTGAGAACTCTGCCACAGCAGCCGCTTACTCTGCTCATTTGGCAGAACAGCAAGCAGACCGTGCGGAACGTGAGGCAGACAAGCTGGAAAATTACAATGGATTGGCTGGTGCAATTGATAAGGTAGATGGAACCAATGTGTACTGGAAAGGAAATATTCACGCTAACGGGCGCCTTTACCTGACCACAAACGGTTTTGAc                                                                                                                                                                                                                                                                                          <  1:84417/301‑1 (MQ=255)`  
                    `gggTTTCCGAGACGAAGCCAAGCGGTTCAAGAATACGGCTGGTCAATACGCTACATCTGCTGGGAACTCTGCTTCCGCTGCGCATCAATCTGAGGTAAACGCTGAGAACTCTGCCACAGCAGCCGCTAACTCTGCTCATTTGGCAGAACAGCAAGCAGACCGTGCGGAACGTGAGGCAGACAAGCTGGAAAATTACAATGGATTGGCTGGTGCAATTGATAAGGTAGATGGAACCAATGTGTACTGGAAAGGAAATATTCACGCTAACGGGCGCCTTTACCTGACCACAAACGGTTTTGAc                                                                                                                                                                                                                                                                                          >  1:595053/1‑301 (MQ=255)`  
                     `ggTTTCCGAGATGAAGCCAAGCGGTTCAAGAATACGGCTGGTCAATACGCTACATCTGCTGGGAACTCTGCTTCCGCTGCGCATCAATCTGAGGTAAACGCTGAGAACTCTGCCACAGCAGCCGCTAACTCTGCTCATTTGGCAGAACAGCAAGCAGACCGTGCGGAACGTGAGGCAGACAAGCTGGAAAATTACAATGGATTGGCTGGTGCAATTGATAAGGTAGATGGAACCAATGTGTACTGGAAAGGAAATATTCACGCTAACGGGCGCCTTTACATGACCACAAACGGTTTTGACt                                                                                                                                                                                                                                                                                         >  1:51257/1‑301 (MQ=255)`  
                             `agaCGAAGCCAAGCGGTTCAAGAATACGGCTGGTCAATACGCTACATCTGCTGGGAACTCTGCTTCCGCTGCGCATCAATCTGAGGTAAACGCTGAGAACTCTGCCACAGCAGCCGCTAACTCTGCTCATTTGGCAGAACAGCAAGCAGACCGTGCGGAACGTGAGGCAGACAAGCTGGAAAATTACAATGGATTGGCTGGTGCAATTGATAAGGTAGATGGAACCAATGTGTACTGGAAAGGAAATATTCACGCTAACTGGCGCCTTTACATGACCACAACCGGTTTTGAATGTGGCCAg                                                                                                                                                                                                                                                                                 >  1:762042/1‑301 (MQ=255)`  
                             `agaCGAAGCCAAGCGGTTCAAGAATACGGCTGGTCAATACGCTACATCTGCTGGGAACTCTGCTTCCGCTGCGCATCAATCTGAGGTAAACGCTGAGAACTCTGCCACAGCAGCCGCTAACTCTGCTCATTTGGCAGAACAGCAAGCAGACCGTGCGGAACGTGAGGCAGACAAGCTGGAAAATTACAATGGATTGGCTGGTGCAATTGATAAGGTAGATGGAACCAATGTGTACTGGAAAGGAAATATTCACGCTAACGGGCGCCTTTACCTGACCACAAACGGTTTTGACTGTGGCCAg                                                                                                                                                                                                                                                                                 >  1:527474/1‑301 (MQ=255)`  
                                 `gAAGCCAAGCGTGTCAAGAATACGGCTGGTCAATACGCTACATCTGCTGTGAACTCTGCTTCCGCTGCGCATCAATCTGAGGTAAACGCTGAGAACTCTGCCACAGCAGCCGCGAACTCTGCTCATTTGGCAGAACAGCAAGCAGACCGTGCGGAACGTGAGGCAGACAAGCTGGAAAATTACAATGGATTGGCTGGTGCAATTGATAAGGTAGATGGAACCAATGTGTACTGGAAAGGAAATATTCACGATAACGGGCGCCTTTACCTGACCACAAACGGTTTTGACTGTGGCCAGTATc                                                                                                                                                                                                                                                                             <  1:92711/301‑1 (MQ=255)`  
                                    `tgtataagagacaggAATACGGCTGGTCAATACGCTACATCTGCTGGGAACTCTGCTTCCGCTGCGCATCAATCTGAGGTAAACGCTGAGAACTCTGCCACAGCAGCCGCTAACTCTGCTCATTTGGCAGAACAGCAAGCAGACCGTGCGGAACGTGAGGCAGACAAGCTGGAAAATTACAATGGATTGGCTGGTGCAATTGATAAGGTAGATGGAACCAATGTGTACTGGAAAGGAAATATTCACGCTAACGGGCGCCTTTACATGACCACAAACGGTTTTGACTGTGGCCAGTATCAAc                                                                                                                                                                                                                                                                          <  1:105823/287‑1 (MQ=255)`  
                                    `gCCAAGTGGTTAAAGAATATGGCTGGTCAATACGCTACATCTGCTGGGAACTCTGCTTCCGCTGCGCATCAATCTGAGGTAAACGCTGAGAACTCTGCCACAGCAGCCGCTAACTCTGCTCATTTGGCAGAACAGCAAGCAGACCGTGCGGAACGTGAGGCAGACAAGCTGGAAAATTACAATGGATTGGCTGGTGCAATTGATAAGGTAGATGGAACCAATGTGTACTGGAAAGGAAATATTCACGCTAACGGGCGCCTTTACATGACCACAAACGGTTTGGAATATGGCCAGTATCAAc                                                                                                                                                                                                                                                                          <  1:740033/301‑1 (MQ=255)`  
                                           `ggTTCAAGAATACGGCTGGTCAATACGCTACATCTGCTGGGAACTCTGCTTCCGCTGCGCATCAATCTGAGGTAAACGCTGAGAACTCTGCCACAGCAGCCGCTAACTCTGCTCATTTGGCAGAAAAGCAAGCAGACCGTGCGGAACGTGAGGCAGACAAGCTGGAAAATTACAATGGATTGGCTGGTGCAATTGATAAGGTAGATGGAACCAATGTGTACTGGAAAGGAAATATTCACGCTAACGGGCGCCTTTACCTGACCACAAACGGTTTTGACTGTGGCCAGTATCAACAGTTCtt                                                                                                                                                                                                                                                                   <  1:790466/301‑1 (MQ=255)`  
                                            `gTTCAAGACTACGGCTGGTCAATACGCTACATCTGCTGGGAACTCTGCTTCCGCTGCGCATCAATCTGAGGTAAACGCTGAGAACTCTGCCACAGCAGCCGCTAACTATGCTCATTTGGCAGAACAGCAAGCAGACCGTGCGGAACGTGAGGCAGACAAGCTGGAAAATTACAATGGATTGGCTGGTGCAATTGATAAGGTAGATGGAACCAATGTGTACTGGAAAGGAAATATTCACGCTAACGGGCGCCTTTACATGACCACAAACGGTTTTGACTGTGGCCAGTATCAACAGTTAttt                                                                                                                                                                                                                                                                  >  1:141178/1‑301 (MQ=255)`  
                                            `gTTCAAGAATACGGCTGGTCAATACGCTACATCTGCTGGGAACTCTGCTTCCGCTGCGCATCAATCTGAGGTAAACGCTGAGAACTCTGCCACAGCAGCCGCTAACTCTGCTCATTTGGCAGAACAGCAAGCAGACCGTGCGGAACGTGAGGCAGACAAGCTGGAAAATTACAATGGATTGGCTGGTGCAATTGATAAGGTAGATGGAACCAATGTGTACTGGAAAGGAAATATTCACGCTAACGGGCGCCTTTACATGACCACAAACGGTTTTGACTGTGGCCAGTATCAACAGTTCttt                                                                                                                                                                                                                                                                  >  1:112830/1‑301 (MQ=255)`  
                                              `acaggAATACGGCTGGTCAATACGCTACATCTGCTGGGAACTCTGCTTCCGCTGCGCATCAATCTGAGGTAAACGCTGAGAACTCTGCCACAGCATCCGCTAACTATGCTCATTTGGCAGAACAGCAAGCAGACCGTGCGGAACGTGAGGCAGACAAGCTGGAAAATTACAATGGATTGGCTGGTGCAATTGATAAGGTAGATGGAACCAATGTGTACTGGAAAGGAAATATTCACGCTAACGGGCGCCTTTACATGACCACAAACGGTTTTGACTGTGGCCAGTATCAACAGTTCTTtgg                                                                                                                                                                                                                                                                <  1:580529/297‑1 (MQ=255)`  
                                               `cAAGAATACGGCTGGTCAATACGCTACATCTGCTGGGAACTCTGCTTCCGCTGCGCATCAATCTGAGGTAAACGCTGAGAACTCTGCCACAGCATCCGCTAACTCTGCTCATTTGGCAGAACAGCAAGCAGACCGTGCGGCACGTGAGGCAGACAAGCTGGAAAATTACAATGGATTGGCTGGTGCAATTGATAAGGTAGATGGAACCAATGTGTACTGGAAAGGAAATATTAACGCTAACGGGCGCCTTTACATGACCACAAACGGTTTTGACTGTGGCCAGTATCAACAGTTCTTtggg                                                                                                                                                                                                                                                               >  1:639482/1‑300 (MQ=255)`  
                                                 `aGAATACGGCTGGTCAATACGCTACATCTGCTGGGAACTCTGCTTACGATGCGCATCAATCTGAGGTAAACGCTGAGAACTCTGCCACAGCAGCCGCTAACTATGCTCATTTGGCAGAACAGCAAGCAGACCGTGCGGAACGTGAGGCAGACAAGCTGGAAAATTACAATGGATTGGCTGGTGCAATTGATAAGGTAGATGGAACCAATGTGTACTGGAAAGGAAATATTCACGCTAACGGGCGCCTTTACATGACCACAAACGGTTTTGACTGTGGCCAGTATCAACAGTTCTTtggtgg                                                                                                                                                                                                                                                             <  1:135164/301‑1 (MQ=255)`  
                                                  `gAATATGGCTGGTCAATACGCTACATCTGCTGGGAACTCTGCTTCCGCTGCGCATCAATCTGAGGTAAACGCTGAGAACTCTGCCACAGCATCCGCTAACTATGCTCATTTGGCAGAACAGCAAGCAGACCGTGCGGAACGTGAGGCAGACAAGCTGGAAAATTACAATGGATTGGCTGGTGCAATTGATAAGGTAGATGGAACCAATGTGTACTGGAAAGGAAATATTCACGCTAACGGGCGCCTTTACATGACCACAAACGGTTTTGACTGTGGCCAGTATCAACAgctgtctcttata                                                                                                                                                                                                                                                            >  1:633589/1‑289 (MQ=255)`  
                                                  `gAATACGGCTGGTCAATACGCTACATCTGCTGGGAACTCTGCTTCCGCTGCGCATCAATCTGAGGTAAACGCTGAGAACTCTGCCACAGCATCCGCTAACTCTGCTCATTTGGCAGAACAGCACGCAGACCGTGCGGAACGTGAGGCAGACAAGCTGGAAAATTACAATGGATTGGCTGGTGCAATTGATAAGGTAGATGGAACCAATGTGTACTGGAAAGGAAATATTCACGCTAACGGGCGCCTTTACATGACCACAAACGGTTTTGACTGTGGCCAGTATCAACAGTTCTTTggtggt                                                                                                                                                                                                                                                            >  1:268843/1‑301 (MQ=255)`  
                                                            `ggTCAATACGCTACATCTGCTGGGAACTCTGCTTCCGCTGCGCATCAATCTGAGGTAAACGCTGAGAACTCTGCCACAGCATCCGCTAACTCTGCTCATTTGGCAGAACAGCAAGCAGACCGTGCGGAACGTGAGGCAGACAAGCTGGAAAATTACAATGGATTGGCTGGTGCAATTGATAAGGTAGATGGAACCAATGTGTACTGGAAAGGAAATATTCACGCTAACGGGCGCCTTTACCTGACCACAAACGGTTTTGACTGTGGCCAGTATCAACAGTTCTTTGGTGGTGTCACTAATc                                                                                                                                                                                                                                                  >  1:688156/1‑301 (MQ=255)`  
                                                            `ggTCAATACGCTACATCTGCTGGGAACTCTGCTTCCGCTGCGCATCAATCTGAGGTAAACGCTGAGAACTCTGCCACAGCATCCGCTAACTATGCTCATTTGGCAGAACAGCAAGCAGACCGTGCTGAACGTGAGGCAGACAAGCTGGAAAATTACAATGGATTGGCTGGTGCAATTGATAAGGTAGATGGAACCAATGTGTACTGGAAAGGAAATATTCACGCTAACGGGCGCCTTTACCTGACCACAAACGGTTTTGACTGTGGCCAGTATCAACAGTTCTTTGGTGGTGTCACTAATc                                                                                                                                                                                                                                                  >  1:100093/1‑301 (MQ=255)`  
                                                            `ggTCAATACGCTACATCTGCTGGGAACTCTGCTTCCGCTGCGCATCAATCTGAGGTAAACGCTGAGAACTCTGCCACAGCAGCCACTAACTCTGCTCATTTGGCAGAACAGCAAGCAGACCGTGCGGAACGTGAGGCAGACAAGCTGGAAAATTACAATGGATTGGCTGGTGCAATTGATAAGGTAGATGGAACCAATGTGTACTGGAAAGGAAATATTCACGCTAACGGGCGCCTTTACCTGACCACAAACGGTTTTGACTGTGGCCAGTATCAACAGTTCTTTGGTGGTGTCACTAATc                                                                                                                                                                                                                                                  >  1:22740/1‑301 (MQ=255)`  
                                                             `gtaaaTACGCTACATCTGCTGGGAACTCTGCTTCCGCTGCGCATCAATCTGAGGTAAACGCTGAGAACTCTGCCACAGCAGCCGCTAACTCTGCTCATTTGGCAGAACAGCAAGCAGACCGTGCGGAACGTGAGGCAGACAAGCTGGAAAATTACAATGGATTGGCTGGTGCAATTGATAAGGTAGATGGAACCAATGTGTACTGGAAAGGAAATATTCACGCTAACGGGCGCCTTTACCTGACCACAAACGGTTTTGACTGTGGCCAGTATCAACAGTTCTTTGGTGGTGTCACTAATCg                                                                                                                                                                                                                                                 <  1:768504/298‑1 (MQ=255)`  
                                                               `aaaTACGCTACATCTGCTGGGAACTCTGCTTCCGCTGCGCATCAATCTGAGGTAAACGCTGAGAACTCTGCCACAGCATCCGCTAACTATGCTCATTTGGCAGAACAGCAAGCAGACCGTGCGGAACGTGAGGCAGACAAGCTGGAAAATTACAATGGATTGGCTGGTGCAATTGATAAGGTAGATGGAACCAATGTGTACTGGAAAGGAAATATTCACGCTAACGGGCGCCTTTACCTGACCACAAACGGTTTTGACTGTGGCCAGTATCAACAGTTCTTTGGTGGTGTCACTAATCGtt                                                                                                                                                                                                                                               <  1:211373/300‑1 (MQ=255)`  
                                                                 `aTACGCTACATCTGCTGGGAACTCTGCTTCCGCTGCGCATCAATCTGAGGTAAACGCTGAGAACTCTGCCACAGCAGCCGCTAACTCTGCTCATTTGGCAGAACAGCAAGCAGACCGTGCGGAACGTGAGGCAGACAAGCTGGAAAATTACAATGGATTGGCTGGTGCAATTGATAAGGTAGATGGAACCAATGTGTACTGGAAAGGAAATATTCACGCTAACGGGCGCCTTTACCTGACCACAAACGGTTTTGACTGTGGCCAGTATCAACAGTTCTTTGGTGGTGTCACTAATCGTTAc                                                                                                                                                                                                                                             >  1:307773/1‑301 (MQ=255)`  
                                                                   `aCGCTACCTCTGCTGGGAACTCTGCTTCCGCTTCGCATCAATCTTAGGTAAACGCTGAGAACTCTGCCACAGCATCCGCTAACTATGCTCATTTGGCAGAACAGCAAGCAGACCGTGCGGAACGTGCGGCAGACAAGCTGGAAAATTACAATGGATTGGCTGGTGCAATTGATAAGGTAGATGGAACCAATGTGTACTGGAAAGGAAATATTCACGCTAACGGGCGCCTTTACATGACCACAAACGGTTTTGACTGTGGCCAGTATCAACAGTTCTTTGGTGGTGTCACTAATCGTTActc                                                                                                                                                                                                                                           <  1:448355/301‑1 (MQ=255)`  
                                                                   `aCGCTACATCTGCTGGGAACTCTGCTTCCGCTGCGCATCAATCTGAGGTAAACGCTGAGAACTCTGCCACAGCATCCGCTAACTCTGCTCATTTGGCAGAACAGCAAGCAGACCGTGCGAAACGTGAGGCAGACAAGCTGGAAAATTACAATGGATTGGCTGGTGCAATTGATAAGGTAGATGGAACCAATGTGTACTGGAAAGGAAATATTCACGCTAACGGGCGCCTTTACCTGACCACAAACGGTTTTGACTGTGGCCAGTATCAACAGTTCTTTGGTGGTGTCACTAATCGTTActc                                                                                                                                                                                                                                           >  1:782618/1‑301 (MQ=255)`  
                                                                    `cgcatcATCGGCTGGGAACTCTTGTTCCGCTGCGCATCAATCTGAGGTAAACGCTGAGAACTCTGCCACAGCAGCCGCTAAATATGCTCATTTGGCAGAACAGCAAGCAGACCGTGCGGAACGTGAGGCAGACAAGCTGGAAAATTACAATTGATTGGCTCGTGCAATTGATAAGGT‑GATGTAACCAATGTGTACTGGAAAGGAAATATTCACGCTAACGGACTCCTATACATGACCACAAACGGTTTTGACTGTGGCCAGTATCAACAGTTCTTTGGTGGTGTCACTAATCGTTACTCTg                                                                                                                                                                                                                                         <  1:814648/296‑1 (MQ=255)`  
                                                                           `tCTGCTGGGAACTCTGCTTCCGCTGCGCATCAATCTGAGGTAAACGCTGAGAACTCTGCCACAGCAGCCGCTAACTCTGCTCATTTGGCAGAACAGCAAGCAGACCGTGCGGAACGTGAGGCAGACAAGCTGGAAAATTACAATGGATTGGCTGGTGCAATTGATAAGGTAGATGGAACCAATGTGTACTGGAAAGGAAATATTCACGCTAACGGGCGCCTTTACCTGACCACAAACGGTTTTGACTGTGGCCAGTATCAACAGTTCTTTGGTGGTGTCACTAATCGTTACTCTGTCATgg                                                                                                                                                                                                                                   <  1:718691/301‑1 (MQ=255)`  
                                                                             `tgctgGGAACTCTGCTTCCGCTGCGCATCAATCTGAGGTAAACGCTGAGAACTCTGCCACAGCAGCCGCTAACTCTGCTCATTTGGCAGAACAGCAAGCAGACCGTGCGGAACGTGAGGCAGACAAGCTGGAAAATTACAATGGATTGGCTGGTGCAATTGATAAGGTAGATGGAACCAATGTGTACTGGAAAGGAAATATTCACGCTAACGGGCGCCTTTACATGACCACAAACGGATTTGACTGTGGCCAGTATCAACAGTTCTTTGGTGGTGTCACTAATCGTTACTCTGTCATGGAg                                                                                                                                                                                                                                 <  1:733913/301‑1 (MQ=255)`  
                                                                                  `ggAACTCTGCTTCCGCTGCGCATCAATCTGAGGTAAACGCTGAGAACTCTGCCACAGCAGCCGCTAACTCTGCTCATTTGGCAGAACAGCAAGCAGACCGTGCGGAACGTGCGGCAGACAAGCTGGAAAATTACAATGGATTGGCTGGTGCAATTGATAAGGTAGATGGAACCAATGTGTACTGGAAAGGAAATATTCACGCTAACGGGCGCCTTTACATGACCACAAACGGTTTTGACTGTGGCCAGTATCAACAGTTCTTTGGTGGTGTCACTAATCGTTACTCTGTCATGGAGTgggg                                                                                                                                                                                                                            <  1:171343/301‑1 (MQ=255)`  
                                                                                       `tctGCTTCCGCTGCGCATCAATCTGAGGTAAACGCTGAGAACTCTGCCACAGCATCCGCTAACTCGGCTCATTTGGCAGAACAGCAAGCAGACCGTGCGGAACGTGAGGCAGACAAGCTGGAAAATTACAATGGATTGGCTGGTGCAATTGATAAGGTAGATGGAACCAATGTGTACTGGAAAGGAAATATTCACGCTAACGGGCGCCTTTACCTGACCACAAACGGTTTTGACTGTGGCCAGTATCAACAGTTCTTTGGTGGTGTCACTAATCGTTACTCTGTCATGGAGTGGGGAGATg                                                                                                                                                                                                                       <  1:72634/301‑1 (MQ=255)`  
                                                                                       `agatgtgtataagagacagaaTCTGAGGTAAACGCTGAGAACTCTGCCACAGCAGCCGCTAACTCTGCTCATTTGGCAGAACAGCAAGCAGACCGTGCGGAACGTGAGGCAGACAAGCTGGAAAATTACAATGGATTGGCTGGTGCAATTGATAAGGTAGATGGAACCAATGTGTACTGGAAAGGAAATATTCACGCTAACGGGCGCCTTTACCTGACCACAAACGGTTTTGACTGTGGCCAGTATCAACAGTTCTTTGGTGGTGTCACTAATCGTTACTCTGTCATGGAGTGGGGAGATg                                                                                                                                                                                                                       <  1:277870/282‑1 (MQ=255)`  
                                                                                        `ctGCTTCCGCTGCGCATCAATCTGAGGTAAACGCTGAGAACTCTGCCACAGCAGCCGCTAACTCTGCTCATTTGGCAGAACAGCAAGCAGACCGTGCGGAACGTGAGGCAGACAAGCTGGAAAATTACAATGGATTGGCTGGTGCAATTGATAAGGTAGATGGAACCAATGTGTACTGGAAAGGAAATATTCACGCTAACGGGCGCCTTTACCTGACCACAAACGGTTTTGACTGTGGCCAGTATCAACAGTTCTTTGGTGGTGTCACTAATCGTTACTCTGTCATGGAGTGGGGAGATga                                                                                                                                                                                                                      >  1:672297/1‑301 (MQ=255)`  
                                                                                          `gCTTCCGCTGCGCATCAATCTGAGGTAAACGCTGAGAACTCTGCCACAGCAGCCGCTAACTCTGCTCATTTGGCAGAACAGCAAGCAGACCGTGCGGAACGTGAGGCAGACAAGCTGGAAAATTACAATGGATTGGCTGGTGCAATTGATAAGGTAGATGGAACCAATGTGTACTGGAAAGGAAATATTCACGCTAACGGGCGCCTTTACATGACCACAAACGGTTTTGACTGTGGCCAGTATCAACAGTTCTTTTGTGGTGTCACTAATCGTTACTCTGTCATGGAGTGGGGAGATgaga                                                                                                                                                                                                                    >  1:653330/1‑301 (MQ=255)`  
                                                                                                   `gcgcATCAATCTGAGGTAAACGCTGAGAACTCTGCCACAGCATCCGCTAACTCTGCTCATTTGGCAGAACAGCAAGCAGACCGTGCGGAACGTGAGGCAGACAAGCTGGAAAATTACAATGGATTGGCTGGTGCAATTGATAAGGTAGATGGAACCAATGTGTACTGGAAAGGAAATATTCACGCTAACGGGCGCCTTTACCTGACCACAAACGGTTTTGACTGTGGCCAGTATCAACAGTTCTTTGGTGGTGTCACTAATCGTTACTCTGTCATGGAGTGGGGAGATGAGAACGGATGGc                                                                                                                                                                                                           >  1:234196/1‑301 (MQ=255)`  
                                                                                                    `cgcATCAATCTGAGGTAAACGCTGAGGACTCTGCCACAGCAGCCGCTAACTCTGCTCATTTGGCAGAACAGCAAGCAGACCGTGCGGAACGTGAGGCAGACAAGCTGGAAAATTACAATGGATTGGCTGGTGCAATTGATAAGGTAGATGGAACCAATGTGTACTGGAAAGGAAATATTCACGCTAACGGGCGCCTTTACCTGACCACAAACGGTTTTGACTGTGGCCAGTATCAACAGTTCTTTGGTGGTGTCACTAATCGTTACTCTGTCATGGAGTGGGGAGATGAGAACGGATGGCt                                                                                                                                                                                                          >  1:625626/1‑301 (MQ=255)`  
                                                                                                        `tCAATCTGAGGTAAACGCTGAGAACTCTGCCACAGCAGCCGCTAACTCTGCTCATTTGGCAGAACAGCAAGCAGACCGTGCGGAACGTGAGGCAGACAAGCTGGAAAATTACAATGGATTGGCTGGTGCAATTGATAAGGTAGATGGAACCAATGTGTACTGGAAAGGAAATATTCACGCTAACGGGCGCCTTTACCTGACCACAAACGGTTTTGACTGTGGCTAGTATCAACAGTTCTTTGGTGGTGTCACTAATCGTTACTCTGTCATGGAGTGGGGAGATGAGAACGGATGGCTGatg                                                                                                                                                                                                      <  1:565682/301‑1 (MQ=255)`  
                                                                                                          `aaTCTGAGGTAAACTCTGAGAACTCTGCCACAGCAGCCGCTAACTCTGCTCATTTGGCAGAACAGCAAGCAGACCGTGCGGAACGTGAGGCAGACAAGCTGGAAAATTACAATGGATTGGCTGGTGCAATTGATAAGGTAGATGGAACCAATGTGTACTGGAAAGGAAATATTCACGCTAACGGGCGCCTTTACATGACCACAAACGGTTTTGACTGTGGCCAGTATCAACAGTTCTTTGGTGGTGTCACTAATCGTTACTCTGTCATGGAGTGGGGAGATGAGAACGGATGGCTGatgta                                                                                                                                                                                                    <  1:798542/301‑1 (MQ=255)`  
                                                                                                           `aTCTGAGGTAAACGCTGAGAACTCTGCCACAGCATCCGCTAACTCTGCTCATTTGGCAGAACAGCAAGCAGACCGTGCGGAACGTGAGGCAGACAAGCTGGAAAATTACAATGGATTGGCTGGTGCAATTGATAAGGTAGATGGAACCAATGTGTACTGGAAAGGAAATATTCACGCTAACGGGCGCCTTTACCTGACCACAAACGGTTTTGACTGTGGCCAGTATCAACAGTTCTTTGGTGGTGTCACTAATCGTTACTCTGTCATGGAGTGGGGAGATGAGAACGGATGGCTGatgtat                                                                                                                                                                                                   >  1:587512/1‑301 (MQ=255)`  
                                                                                                               `gAGGTAAACGCTGAGAACTCTGCCACAGCATCCGCTATCTTTGCTCATTTGGCAGAACAGCAAGCAGACCGTGCGGAACGTGAGGCAGACAAGCTGGAAAATTACAATGGATTGGCTGGTGCAATTGATAAGGTAGATGGAACCAATGTGTACTGGAAAGGAAATATTCACGCTAACGGGCGCCTTTACATGACCACAAACGGTTTTGACTGTGGCCAGTATCAACAGTTCTTTGGTGGTGTCACTAATCGTTACTCTGTCATGGAGTGGGGAGATGAGAACGGATGGCTGatgtactgtc                                                                                                                                                                                               >  1:774757/1‑296 (MQ=255)`  
                                                                                                               `gAGGTAAACGCTGAGAACTCTGCCACAGCAGCCGCTAACTCTGCTCATTTGGCAGAACAGCAAGCAGACCGTGCGGAACGTGAGGCAGACAAGCTGGAAAATTACAATGGATTGGCTGGTGCAATTGATAAGGTAGATGGAACCAATGTGTACTGGAAAGGAAATATTCACGCTAACGGGCGCCTTTACCTGACCACAAACGGTTTTGACTGTGGCCAGTATCAACAGTTCTTTGGTGGTGTCACTAATCGTTACTCTGTCATGGAGTGGGGAGATGAGAACGGATGGCTGactgtctctt                                                                                                                                                                                               >  1:241541/1‑292 (MQ=255)`  
                                                                                                               `gAGGTAAACGCTGAGAACTCTGCCACAGCAGCCGCTAACTCTGCTCATTTGGCAGAACAGCAAGCAGACCGGGCGGAACGTGAGGCAGACAAGCTGGAAAATTACAATGGATTGGCTGGTGCAATTGATAAGGTAGATGGAACCAATGTGTACTGGAAAGGAAATATTCACGCTAACGGGCGCCTTTACCTGACCACAAACGGTTTTGACTGTGGCCAGTATCAACAGTTCTTTGGTGGTGTCACTAATCGTTACTCTGTCATGGAGTGGGGAGATGAGAACGGATGGCTGATGTATGTTc                                                                                                                                                                                               <  1:156491/301‑1 (MQ=255)`  
                                                                                                               `agggTAAACGCTGAGAACTCTGCCACAGCAGCCGCTAACTCTGCTCATTTGGCAGAACAGCACGCAGACCGTGCGGAACGTGAGGCAGACAAGCTGGAAAATTACAATGGATTGGCTGGTGCAATTGATAAGGTAGATGGAACCAATGTGTACTGGAAAGGAAATATTCACGCTAACGGGCGCCTTTACATGACCACAAACGGTTTTGACTGTGGCCAGTATCAACAGTTCTTTGGTGATGTCACTAATCGTTACTCTGTCATGGAGTGGGGAGATGAGAACGGATGGCTGATGTATGTTc                                                                                                                                                                                               <  1:402634/299‑1 (MQ=255)`  
                                                                                                                 `ggTAAACGCTGAGAACTCTGCCACAGCAGCCGCTAACTCTGCTCATTTGGCAGAACAGCAAGCAGACCGTGCGGAACGTGCGGCAGACAAGCTGGAAAATTACAATGGATTGGCTGGTGCAATTGATAAGGTAGATGGAACCAATGTGTACTGGAAAGGAAATATTCACGCTAACGGGCGCCTTTACATGACCACAAACGGTTTTGACTGTGGCCAGTATCAACAGTTCTTTGGTGGTGTCACTAATCGTTACTCTGTCATGGAGTGGGGAGATGAGAACGGCTGGCTGATGTATGTTCaa                                                                                                                                                                                             >  1:666430/1‑301 (MQ=255)`  
                                                                                                                        `gCTGAGAACTCTGCCACAGCAGCCGCTAACTCTGCTCATTTGGCAGAACAGCAAGCAGACCGTGCGGAACGTGAGGCAGACAAGCTGGAAAATTACAATGGATTGGCTGGTGCAATTGATAAGGTAGATGGAACCAATGTGTACTGGAAAGGAAATATTCACGCTAACGGGCGCCTTTACCTGACCACAAACGGTTTTGACTGTGGCCAGTATCAACAGTTCTTTGGTGGTGTCACTAATCGTTACCCTGTCATGGAGTGGGGAGATGAGAACGGATGGCTAATGGATGTTCAACGtctgg                                                                                                                                                                                      >  1:64686/1‑297 (MQ=255)`  
                                                                                                                        `gCTGAGAACTCTGCCACAGCAGCCGCTAACTATGCTCATTTGGCAGAACAGCAAGCAGACCGTGCGGAACGTGAGGCAGACAAGCTGGAAAATTACAATGGATTGGCTGGTGCAATTGATAAGGTAGATGGAACCAATGTGTACTGGAAAGGAAATATTCACGCTAACGGGCGCCTTTACATGACCACAAACGGTTTTGACTGTGGCCAGTATCAACAGTTCTTTGGTGGTGTCACTAATCGTTACTCTGTCATGGAGTGGGGAGATGAGAACGGATGGCTGATGTATGTTCAACGTagag                                                                                                                                                                                      <  1:462880/301‑1 (MQ=255)`  
                                                                                                                             `gaACTCTGCCACAGCATCCGCTAACTCTGCTCATTTGGCAGAACAGCAAGCAGACCGTGCGGAACGTGAGGCAGACTAGCTGGAAAATTACAATGGATTTGCTGTTGCAATTGTTATGGTAGATGGATCCTATGTGTACTGTTAAGGTAATATTCACGCTAACGGGCGCCTTTACCTGACCACAACCTGTTTTGACTGTGTCCATTATCAACAGTTCTTTGGTGGTTTCACTAATCGTTACTCTGTCATTGAGTGTGGAGATGATTACGGATTGCTGATGTTTGTTCAACGtctgtctctt                                                                                                                                                                                 >  1:261880/1‑292 (MQ=255)`  
                                                                                                                               `ataagagacaggCATCCGCTAACTTTGCTCATTTGGCAGAACAGCAAGCAGACCGTGCGGAACGTGAGGCAGACAAGCTGGAAAATTACAATGGATTGGCTGGTGCAATTGATAAGGTAGATGGAACCAATGTGTACTGGAAAGGAAATATTCACGCTAACGGGCGCCTTTACATGACCACAAACGGTTTTGACTGTGGCCAGTATCAACAGTTCTTTGGTGGTGTCACTAATCGTTACTCTGTCATGGAGTGGGGAGATGAGAACGGATGGCTGATGTATGTTCAACGTAGAGAGTGGac                                                                                                                                                                               <  1:339205/290‑1 (MQ=255)`  
                                                                                                                                `ctctGCCACAGCATCCGCTAACTTTGCTCATTTGGCAGAACAGCAAGCAGACCGTGCGGAACGTGAGGCAGACAAGCTGGAAAATTACAATGGATTGGCTGGTGCAATTGATAAGGTAGATGGAACCAATGTGTACTGGAAAGGAAATATTCACGCTAACGGGCGCCTTTACATGACCACAAACGGTTTTGACTGTGGCCAGTATCAACAGTTCTTTGGTGGTGTCACTAATCGTTACTCTGTCATGGAGTGGGGAGATGAGAACGGATGGCTGATGTATGTTCAACGTAGAGAGTGGaca                                                                                                                                                                              >  1:535964/1‑301 (MQ=255)`  
                                                                                                                                `ctatGCCACAGCAGCCGCTAACTCTGCTCATTTGGCAGAACAGCAAGCAGACCGTGCGGAACGTGAGGCAGACAAGCTGGATAATTACAATGGATTGGCTGGTGCAATTGATAATCTAGATGGAACAATTGTGTACTGTAAAGGACATATTCACGCTAACGGACGCCTTTACATTCCCACAACCGGTTTTGACTGTGGCCAGTATCAACAGTTCTTTGGTGGTGTCACTAATCGTTACTCTGTCATGGAGTGGGGTGATGATAACGGATGGCTGATGTATGTTCAACGTAGAgtgtctttt                                                                                                                                                                              >  1:808154/4‑296 (MQ=255)`  
                                                                                                                                  `ctGCCACAGCATCCGCTAACTATGCTCATTTGGCAGAACAGCAAGCAGACCGTGCGGAACGTGAGGCAGACAAGCTGGAAAATTACAATGGATTGGCTGGTGCAATTGATAAGGTAGATGGAACCAATGTGTACTGGAAAGGAAATATTCACGCTAACGGGCGCCTTTACATGACCACAAACGGTTTTGACTGTGGCCAGTATCAACAGTTCTTTGGTGGTGTCACTAATCGTTACTCTGTCATGGAGTGGGGAGATGAGAACGGATGGCTGATGTATGTTCAACGTAGAGAGTGGacact                                                                                                                                                                            >  1:163172/1‑299 (MQ=255)`  
                                                                                                                                        `caGCATCCGCTAACTCTGCTCATTTGGCAGAACAGCAAGCAGACCGTGCGGAACGTGAGGCAGACAAGCTGGAAAATTTCAATGGATTGGCTGGTGCAATTGATAAGGTAGATGGAACCAATGTGTACTGGAAAGGAAATATTCACGCTAACGGGCGCCTTTACCTGACCACAAACGGTTTTGACTGTGGCCAGTATCAACAGTTCTTTGGTGGTGTCACTAATCGTTACTCTGTCATGGAGTGGGGAGATGAGAACGGATGGCTGATGTATGTTCAACGTAGAGAGTGGACAACAGCGAt                                                                                                                                                                      >  1:316179/1‑301 (MQ=255)`  
                                                                                                                                           `cagccGCTAACTCTGCTCATTTGGCAGAACAGCAAGCAGACCGTGCGGAACGTGAGGCAGACAAGCTGGAAAATTACAATGGATTGGCTGGTGCAATTGATAAGGTAGATGGAACCAATGTGTACTGGAAAGGAAATATTCACGCTAACGGGCGCCTTTACATGACCACAAACGGTTTTGACTGTGGCCAGTATCAACAGTTCTTTGGTGGTGTCACTAATCGTTACTCTGTCATGGAGTGGGGAGATGAGAACGGATGGCTGATGTATGTTCAACGTAGAGAGTGGACAACAGCGATAgg                                                                                                                                                                   <  1:204536/298‑1 (MQ=255)`  
                                                                                                                                             `gccGCTAACTCTGCTCATTTGGCAGAACAGCAAGCAGACAGTGCGGAACGTGAGGCAGACAAGCTGGAAAATTACAATGGATTGGCTGGTGCAATTGATAAGGTAGATGGAACCAATGTGTACTGGAAAGGAAATATTCACGCTAACGGGCGCCTTTACATGACCACAAACGGTTTTGACTGTGGCCAGTATCAACAGTTCTTTGGTGGTGTCACTAATCGTTACTCTGTCATGGAGTGGGGAGATGAGAACGGATGGCTGATGTATGTTCAACGTagagctgtctcttatacacatctcc                                                                                                                                                                 >  1:573181/2‑280 (MQ=255)`  
                                                                                                                                                  `tAACTATGCTCATTTGGCAGAAGAGCAAGCAGACCGTGCGGAACGTGAGGCAGACAAGCTGGAAAATTACAATGGATTGGCTGGTGCAAGTGATAAGGTAGATGGAACCAATGTGTACTGGAAAGGAAATATTCACGCTAACGGGCGCCTTTACCTGACCACAAACGGTTTTGACTGTGGCCAGTATCAACAGTTCTTTGGTGGTGTCACTAATCGTTACTCTGTCATGGAGTGGGGAGATGAGAACGGATGGCTGATGTATGTTCAACGTAGAGAGTGGACAACAGCGATAGGCGGTAAc                                                                                                                                                            <  1:70355/301‑1 (MQ=255)`  
                                                                                                                                                      `tataagagacaggCAGAACAGCAAGCAGACCGTGCGGAACGTGAGGCAGACAAGCTGGAAAATTACAATGGATTGGCTGGTGCAATTGATAAGGTAGATGGAACCAATGTGTACTGGAAAGGAAATATTCACGCTAACGGGCGCCTTTACCTGACCACAAACGGTTTTGACTGTGGCCAGTATCAACAGTTCTTTGGTGGTGTCACTAATCGTTACTCTGTCATGGAGTGGGGAGATGAGAACGGATGGCTGATGTATGTTCAACGTAGAGAGTGGACAACAGCGATAGGCGGTAACATcc                                                                                                                                                        <  1:346566/290‑1 (MQ=255)`  
                                                                                                                                                               `ttGGCAGAACAGCAAGCAGACCGTGCGGAACGTGAGGCAGACAAGCTGGAAAATTACAATGGATTGGCTGGTGCAATTGATAAGGTAGATGTAACCAATGTGTACTGGAAAGGAAATATTCACGCTAACGGGCGCCTTTACATGACCACAAACGGTTTTGACTGTGGCCAGTATCAACAGTTCTTTGGTGGTGTCACTAATCGTTACTCTGTCATGGAGTGGGGAGATGAGAACGGATGGCTTATGTATGTTCAACGTAGAGAGTGGACAACAGCGATAGGCGGTAACATCCAGTtagtag                                                                                                                                               <  1:431210/301‑1 (MQ=255)`  
                                                                                                                                                                        `cAGCACGCAGACCGTGCGGAACGTGAGGCAGACAAGCTGGAAAATTACAATGGATTGGCTGGTGCAATTGATAAGGTAGATGGAACCAATGTGTACTGGAAAGGAAATATTCACGCTAATGGGCGCCTTTACATGACCACAAACGGTTTTGACTGTGGCCAGTATCAACAGTTCTTTGGTGGTGTCACTAATCGTTACTCTGTCATGGAGTGGGGAGATGAGAACGGATGGCTGATGTATGTTCAACGTAGAGAGTGGACAACAGCGATAGGCGGTAACATCCAGTTAGTAGTAAACGGAc                                                                                                                                      <  1:286655/301‑1 (MQ=255)`  
                                                                                                                                                                                     `gTGCGGAACGTGAGGCTGACAAGCTGGAAAATTACAATGGATTGGCTGGTGCAATTGATAAGGTAGATGGAACCAATGTGTACTGGAAAGGAAATATTCACGCTAACGGGCGCCTTTACATGACCACAAACGGTTTTGACTGTGGCCAGTATCAACAGTTCTTTGGTGGTGTCACTAATCGTTACTCTGTCATGGAGTGGGGAGATGAGAACGGATGGCTGATGTATGTTCAACGTAGAGAGTGGACAACAGCGATAGGCGGTAACATCCAGTTagtagtctgtctcttatacacatctcc                                                                                                                         >  1:454940/1‑280 (MQ=255)`  
                                                                                                                                                                                            `aCGTGAGGCAGACAAGCTGGAAAATTACAATGGATTGGCTGGTGCAATTGATAAGGTAGATGGAACCAATGTGTACTGGAAAGGAAATATTCACGCTAACGGGCGCCTTTACCTGACCACAAACGGTTTTGACTGTGGCCAGTATCAACAGTTCTTTGGTGGTGTCACTAATCGTTACTCTGTCATGGAGTGGGGAGATGAGAACGGATGGCTGATGTATGTTCAACGTAGAGAGTGGACAACAGCGATAGGCGGTAACATCCAGTTAGTAGTAAACGGACAGATCATCACCCAAGGTGGa                                                                                                                  >  1:424160/1‑301 (MQ=255)`  
                                                                                                                                                                                            `aCGTGAGGCAGACAAGCTGGAAAATTACAATGGATTGGCTGGTGCAATTGATAAGGTAGATGGAACCAATGTGTACTGGAAAGGAAATATTCACGCTAACGGGCGCCTTTACATGACCACAAACGGTTTTGACTGTGGCCAGTATCAACAGTTCTTTGGTGGTGTCACTAATCGTTACTCTGTCATGGAGTGGGGAGATGAGAACGGATGGCTGATGTATGTTCAACGTAGAGAGTGGACAACAGCGATAGGCGGTAACATCCAGTTAGTAGTAAACGGACAGATCATCACCCAAGGTGGa                                                                                                                  >  1:556297/1‑301 (MQ=255)`  
                                                                                                                                                                                                `gAGGCAGACAAGCTGGAAAATTACAATGGATTGGCTGGTGCAATTGATAAGGTAGATGGAACCAATGTGTACTGGAAAGGAAATATTCACGCTAACGGGCGCCTTTACCTGACCACAAACGGTTTTGACTGTGGCCAGTATCAACAGTTCTTTGGTGGTGTCACTAATCGTTACTCTGTCATGGAGTGGGGAGATGAGAACGGATGGCTGATGTATGTTCAACGTAGAGAGTGGACAACAGCGATAGGCGGTAACATCCAGTTAGTAGTAAACGGACAGATCATCACCCAAGGTGGAGCCa                                                                                                              <  1:710532/301‑1 (MQ=255)`  
                                                                                                                                                                                                  `ggCCGACAAGCTGGAAAATTACAATGGATTGGCTGGTGCAATTGATAAGGTAGATGGAACCAATGTGTACTGGAAAGGAAATATTCACGCTAACGGGCGCCTTTACCTGACCACAAACGGTTTTGACTGTGGCCAGTATCAACAGTTCTTTGGTGGTGTCACTAATCGTTACTCTGTCATGGAGTGGGGAGATGAGAACGGATGGCTGATGTATGTTCAACGTAGAGAGTGGACAACAGCGATAGGCGGTAACATCCAGTTAGTAGTAAACGGACAGATCATCACCCAAGGTGGAGCCATg                                                                                                            >  1:245981/1‑301 (MQ=255)`  
                                                                                                                                                                                                              `ggAAAATTACAATGGATTGGCTGGTGCAATTGATAAGGTAGATGGAACCAATGTGTACTGGAAAGGTAATATTCTCGCTAACTGGCGCCTTTACCTGACCTCCATCGGTTTTGACTTTTTCCAGTATCTACTTTTCTTTTGTTTTTTTACTAATCGTTTCTCTGTCATGTATTGTGGTGATGATATCTTATGTATGATGTATGTTCAACGTATTTAGTGGACAACTTTTATTGGCGGTAACATCCAGTTATTATTAAACTCATATTTCATCACCCAAGGTTGAGCCATTACCgtttatctt                                                                                                >  1:478373/1‑293 (MQ=255)`  
                                                                                                                                                                                                                            `gATTGGCTGGTGCAATTGATAAGGTAGATGGAACCAATGTGTACTGGAAAGGAAATATTCACGCTAACGGGCGCCTTTACCTGACCACAAACGGTTTTGACTGTGGCCAGTATCAACAGTTCTTTGGTGGTGTCACTAATCGTTACTCTGTCATGGAGTGGGGAGATGAGAACGGATGGCTGATGTATGTTCAACGTAGAGAGTGGACAACAGCGATAGGCGGTAACATCCAGTTAGTAGTAAACGGACAGATCATCACCCAAGGTGGAGCCATGACCGGTCAGCTAAAAtctgtctctta                                                                                  >  1:519998/1‑291 (MQ=255)`  
                                                                                                                                                                                                                                `ggCTGGTGCAATTGATAAGGTAGATGGAACCAATGTGTACTGGAAAGGAAATTTTCACGCTAACGGGCGCCTTTACATGACCACAAACGGTTTTGACTGTGGCCAGTATCAACAGTTCTTTGGTGGTGTCACTAATCGTTACTCTGTCATGGAGTGGGGAGATGAGAACGGATGGCTGATGTATGTTCAACGTAGAGAGTGGACAACAGCGATAGGCGGTAACATCCAGTTAGTAGTAAACGGACAGATCATCACCCAAGGTGGAGCCATGACCGGTCAGCTAAAATTGCAGAATGGGCAt                                                                              >  1:36043/1‑301 (MQ=255)`  
                                                                                                                                                                                                                                 `gCTGGTGCAATTGATAAGGTAGATGGAACCAATGTGTACTGGAAAGGAAATATTCACGCTAACGGGCGCCTTTACCTGACCACAAACGGTTTTGACTGTGGCCAGTATCAACAGTTCTTTGGTGGTGTCACTAATCGTTACTCTGTCATGGAGTGGGGAGATGAGAACGGATGGCTGATGTATGTTCAACGTAGAGAGTGGACAACAGCGATAGGCGGTAACATCCAGTTAGTAGTAAACGGACAGATCATCACCCAAGGTGGAGCCATGACCGGTCAGCTAAAATTGCAGAATGGGCATg                                                                             <  1:120923/301‑1 (MQ=255)`  
                                                                                                                                                                                                                                    `ggTGCTATTGATTAGGTAGATGGAACCAATGTGTACTGGAAAGGAAATATGCACGCTAACGGGCGCCTTTACCTGACCACAAACGGTTTTGACTGTGGCCAGTATCAACAGTTCTTTGGTGGTGTCACTAATCGTTACTATGTCATGGAGTGGGGAGATGAGAACGGATGGCTGATGTATGTTCAACGTAGAGAGTGGACAACAGCGATAGGCGGTAACATCCAGTTAGTAGTAAACGGACAGATCATCACCCAAGGTGGAGCCATGACCGGTCAGCTAAAATTGCAGAATGGGCATGttc                                                                          <  1:680057/301‑1 (MQ=255)`  
                                                                                                                                                                                                                                           `ttGATAAGGTAGATGGAACCTATGTGTACTGGAAAGGAAATATTCACGCTAACGGGCTCCTTTACCTGACCACAAACGGTTTTGACTGTGGCCAGTATCAACAGTTCTTTGGTGGTGTCACTAATCGTTACTCTGTCATGGAGTGGGGAGATGAGAACGGATGGCTGATGTATGTTCAACGTAGAGAGTGGACAACAGCGATAGGCGGTAACATCCAGTTAGTAGTAAACGGACAGATCATCACCCAAGGTGGAGCCATGACCGGTCAGCTAAAATTGCAGAATGGGCATGTTCTTCAAtt                                                                   <  1:253893/301‑1 (MQ=255)`  
                                                                                                                                                                                                                                               `tAAGGTAGATGGAACCAATGTGTACTGGAAAGGAAATATTCACGCTAACGGGCGCCTTTACATGACCACAAACGGTTTTGACTGTGGCCAGTATCAACAGTTCTTTGGTGGTGTCACTAATCGTTACTCTGTCATGGAGTGGGGAGATGAGAACGGATGGCTGATGTATGTTCAACGTAGAGAGTGGACAACAGCGATAGGCGGTAACATCCAGTTAGTAGTAAACGGACAGATCATCACCCAAGGTGGAGCCATGACCGGTCAGCTAAAATTGCAGAATGGGCATGTTCTTCAATTagag                                                               >  1:755844/1‑301 (MQ=255)`  
                                                                                                                                                                                                                                               `aaaGGTAGATGGAACCAATGTGTACTGGAAAGGAAATATTCACGCTAACGGGCGCCTTTACATGACCACAAACGGTTTTGACTGTGGCCAGTATCAACAGTTCTTTGGTGGTGTCACTAATCGTTACTCTGTCATGGAGTGGGGAGATGAGAACGGATGGCTGATGTATGTTCAACGTAGAGAGTGGACAACAGCGATAGGCGGTAACATCCAGTTAGTAGTAAACGGACAGATCATCACCCAAGGTGGAGCCATGACCGGTCAGCTAAAATTGCAGAATGGGCATGTTCTTCAATTagag                                                               <  1:178056/300‑1 (MQ=255)`  
                                                                                                                                                                                                                                                 `aGGTAGATGGAACCAATGTGTACTGGAAAGGAAATATTCACGCTAACGGGCGCCTTTACCTGACCACAAACGGTTTTGACTGTGGCCAGTATCAACAGTTCTTTGGTGGTGTCACTAATCGTTACTCTGTCATGGAGTGGGGAGATGAGAACGGATGGCTGATGTATGTTCAACGTAGAGAGTGGACAACAGCGATAGGCGGTAACATCCAGTGAGTAGTAAACGGACAGATCATCACCCAAGGTGGAGCCATGACCGGTCAGCTAAAATTGCAGAATGGGCATGTTCTTCAATTAGAGTc                                                             >  1:185914/1‑301 (MQ=255)`  
                                                                                                                                                                                                                                                  `ggTAGATGGAACCAATGTGTACTGGAAAGGAAATATTCACGCTAACGGGCGCCTTTACCTGACCACAAACGGTTTTGACTGTGGCCAGTATCAACAGTTCTTTGGTGGTGTCACTAATCGTTACTCTGTCATGGAGTGGGTAGATTAGAACGGATGGCTGATGTATGTTCAACGTAGAGAGTGGACAACAGCGATAGGCGGTAACATCCAGTTAGTAGTAAACGGACAGATCATCACCCAAGGTGGAGCCATGACCGGTCAGCTAAAATTGCAGAATGGTCATGTTCTTCAAttctgtctc                                                            >  1:438855/1‑294 (MQ=255)`  
                                                                                                                                                                                                                                                          `gAACCAATGTGTACTGGAAAGGAAATATTCACGCTAACGGGCGCCTTTACATGACCACAAACGGTTTTGACTGTGGCCAGTATCAACAGTTCTTTGGTGGTGTCACTAATCGTTACTCTGTCATGGAGTGGGGAGATGAGAACGGAT‑GCTGATGTATGTTCAACGTAGAGAGTGGACAACAGCGATAGGCGGTAACATCCAGTTAGTAGTAAACGGACAGATCATCACCCAAGGTGGAGCCATGACCGGTCAGCTAAAATTGCAGAATGGGCAGGGTCTTCAATTAGAGTCAGCATCCGAc                                                   >  1:541727/1‑301 (MQ=255)`  
                                                                                                                                                                                                                                                               `aaTGTGTACTGGAAAGGAAATATTAACGCTAACGGGCGCCTTTACATGACCACAAACGGTTTTGACTGTGGCCAGTATCAACAGTTCTTTGGTGGTGTCACTAATCGTTACTCTGTCATGGAGTGGGGAGATGAGAACGGATGGCTGATGTATGTTCAACGTAGAGAGTGGACAACAGCGATAGGCGGTAACATCCAGTTAGTAGTAAACGGACAGATCATCACCCAAGGTGGAGCCATGACCGGTCAGCTAAAATTGCAGAATGGGCATGTTCTTCAATTAGAGTCCGCATCCGACAAgg                                               >  1:470209/1‑301 (MQ=255)`  
                                                                                                                                                                                                                                                                  `gtgtACTGGAAAGGAAATATTCACGCTAACGGGCGCCTTTACCTGACCACAAACGGTTTTGACTGTGGCCAGTATCAACAGTTCTTTGGTGGTGTCACTAATCGTTACTCTGTCATGGAGTGGGGAGATGAGAACGGATGGCTGATGTATGTTCAACGTAGAGAGTGGACAACAGCGATAGGCGGTAACATCCAGTTAGTAGTAAACGGACAGATCATCACCCAAGGTGGAGCCATGACCGGTCAGCTAAAATTGCAGAATGGGCATGTTCTTCAATTAGAGTCCGCATCCGACAAGgcgc                                            >  1:485736/1‑301 (MQ=255)`  
                                                                                                                                                                                                                                                                  `gtgtACTGGAAAGGAAATATTCACGCTAACGGGCGCCTTTACATGACCACAAACGGTTTTGACTGTGGCCAGTATCAACAGTTCTTTGGTGGTGTCACTAATCGTTACTCTGTCATGGAGTGGGGAGATGAGAACGGATGGCTGATGTATGTTCAACGTAGAGAGTGGACAACAGCGATAGGCGGTAACATCCAGTTAGTAGTAAACGGACAGATCATCACCCAAGGTGGAGCCATGACCGGTCAGCTAAAATTGCAGAATGGGCATGTTCTTCAATTAGCGTCCGCATCCGACAAGgcgc                                            >  1:549083/1‑301 (MQ=255)`  
                                                                                                                                                                                                                                                                      `aCTGGAAAGGAAATATTCACGCTAACGGGCGCCTTTACCTGACCACAAACGGTTTTGACTGTGGCCAGTATCAACAGTTCTTTGGTGGTGTCACTAATCGTTACTCTGTCATGGAGTGGGGAGATGAGAACGGATGGCTGATGTATGTTCAACGTAGAGAGTGGACAACAGCGATAGGCGGTAACATCCAGTTAGTAGTAAACGGACAGATCATCACCCAAGGTGGAGCCATGACCGGTCAGCTAAAATTGCAGAATGGGCATGTTCTTCAATTAGAGTCCGCATCCGACAAGGCGCACta                                        <  1:65971/301‑1 (MQ=255)`  
                                                                                                                                                                                                                                                                      `aCTGGAAAGGAAATATTCACGCTAACGGGCGCCTTTACATGACCACAAACGGTTTTGACTGTGGCCAGTATCAACAGTTCTTTGGTGGTGTCACTAATCGTTACTCTGTCATGGAGTGGGGAGATGAGAACGGATGGCTGATGTATGTTCAACGTAGAGAGTGGACAACAGCGATAGGCGGTAACATCCAGTTAGTAGTAAACGGACAGATCATCACACAAGGTGGAGCCATGACCGGTCAGCTAAAATTGCAGAATGGGCATGTTCTTCAATTAGAGTCCGCATCCGACAAGGCGCACta                                        <  1:394820/301‑1 (MQ=255)`  
                                                                                                                                                                                                                                                                       `cTGGAAAGGAAATATTCACGCTAACGGGCGCCTTTACCTGACCACAAACGGTTTTGACTGTGGCCAGTATCAACAGTTCTTTGGTGGTGTCACTAATCGTTACTCTGTCATGGAGTGGGGAGATGAGAACGGATGGCTGATGTATGTTCAACGTAGAGAGTGGACAACAGCGATAGGCGGTAACATCCAGTTAGTAGTAAACGGACAGATCATCACCCAAGGTGGAGCCATGACCGGTCAGCTAAAATTGCAGAATGGGCATGTTCTTCAATTAGAGTCCGCATCCGACAAGGCGCACtat                                       >  1:494385/1‑301 (MQ=255)`  
                                                                                                                                                                                                                                                                          `gaaaggaaaTATTCACGCTAACGGGCGCCTTTACCTGACCACAAACGGTTTTGACTGTGGCCAGTATCAACAGTTCTTTGGTGGTGTCACTAATCGTTACTCTGTCATGGAGTGGGGAGATGAGAACGGATGGCTGATGTATGTTCAACGTAGAGAGTGGACAACAGCGATAGGCGGTAACATCCAGTTAGTAGTAAACGGACAGATCATCACCCAAGGTGGAGCCATGACCGGTCAGCTAAAATTGCAGAATGGGCATGTTCTTCAATTAGAGTCCGCATCCGACAAGGCGCACTATAtt                                    <  1:149318/301‑1 (MQ=255)`  
                                                                                                                                                                                                                                                                            `aaggaaaTATTCACGCTAACGGGCGCCTTTACCTGACCACAAACGGTTTTGACTGTTGCCAGTATCAACAGTTCTTTGGTGGTGTCACTAATCGTTACTCTGTCATGGAGTGGGGAGATGAGAACGGATGGCTGATGTATGTTCAACGTAGAGAGTGGACAACAGCGATAGGCGGTAACATCCAGTTAGTAGTAAACGGACAGATCATCACCCAAGGTGGAGCCATGACCGGTCAGCTAAAATTGCAGAATGGGCATGTTCTTCAATTAGAGTCCGCATCCGACAAGGCGCACTATATtct                                  <  1:37674/301‑1 (MQ=255)`  
                                                                                                                                                                                                                                                                            `aaggaaaTATTCACGCTAACGGGCGCCTTTACCTGACCACAAACGGTTTTGACTGTGGCCATTATCAACAGTACGTTGGTGGTGTAACTAATCGTTACTCTGTCATGGAGTGGGGAGATGAGAACGGATGGCTGATGTATGTTAAACGTAGAGAGTGGACAACAGCGATAGGCGGTAACATCCAGTTAGTAGTAAACGGACAGATCATCACCCAAGGTGGAGCCATGAACGGTCAGCTAAAATTGCAGAATGGGCATGTTCTTCAATTAGAGTCCGCATCCGACAAGGCGCACTATATtct                                  <  1:61944/301‑1 (MQ=255)`  
                                                                                                                                                                                                                                                                            `aaggaaaTATTCACGCTAAAGAGCGACTTTACATGACCACAAACGGTTTTAACTGTGGCCAGTATCAACAGTTCTTTGGTGGTGTAACTAATCGTTACTCTGTCATGGAGTTGGGAGATGAGAACGGATGGATGATGTATGTGCAACGTAGAGAGTAGAAAACAGCGATAGGCGGTAACATCCAGTTAGTAGTAAACGGACAGATCATCACCAAAGGTGGAGCCATGACCGGTCAGCTAAAATTGCAGAATGGGCATGTTCTTCAATTAGAGTCCGCATCCGACAAGGCGCACTATATtca                                  <  1:301203/301‑2 (MQ=255)`  
                                                                                                                                                                                                                                                                              `ggaaaTATTCACGCTAACGGGCGCCTTTACCTGACCACAAACGGTTTTTACTGTGGCCAGTATCAACAGTTCTTTGGTGGTGTCACTAATCGTTACTCTGTCATGGAGTGGGGAGATGAGAACGGATGGCTGAGGTATGTTCAACGTAGAGAGTGGACAACAGCGATAGGCGGTAACATCCAGTTAGTAGTAAACGGACAGATCATCACCCAAGGTGGAGCCATGACCGGTCAGCTAAAATTGCAGAATGGTCATGTTCTTCAATTAGAGTCCGCATCCGACAAGGCGCACTATATtctat                                <  1:218402/301‑1 (MQ=255)`  
                                                                                                                                                                                                                                                                              `ggaaaTATTCACGCTAACGGGCGCC‑TTACATGACCACAAACGGTTTTGACTGTGGCCAGTATCAACAGTTCTTTGGTGGTGTCACTAATCGTTACTCTGTCATGGAGTGGGGAGATGAGAACGGATGGCTGATGTATGTTCAACGTAGAGAGTGGACAACAGCGATAGGCGGTAACATCCAGTTAGTAGTAAACGGACAGATCATCACCCAAGGTGGAGCCATGACCGGTCAGCTAAAATTGCAGAATGGGCATGTTTTTCAATTAGAGTCCGCATCCGACAAGGCGCACTATATtatatc                               >  1:747814/1‑301 (MQ=255)`  
                                                                                                                                                                                                                                                                               `gaaaTATTCACGCTAACGGGCGCCTTTACCTGACCACAAACGGTTTTGACTGTGGCCAGTATCAACAGTTCTTTGGTGGTGTCACTAATCGTTACTCTGTCATGGAGTGGGGAGATGAGAACGGATGGCTGATGTATGTTCAACGTAGAGAGTGGACAACAGCGATAGGCGGTAACATCCAGTTAGTAGTAAACGGACAGATCATCACCCAAGGTGGAGCCATGACCGGTCAGCTAAAATTGCAGAATGGGCATGTTCTTCAATTAGAGTCCGCATCCGACAAGGCGCACTATATtctctg                               >  1:417203/1‑298 (MQ=255)`  
                                                                                                                                                                                                                                                                               `gaaaTATTCACGCTAACGGGCGCCTTTACATGACCACAAACGGTTTTGACTGTTGCCAGTATCAACAGTTCTTTGGTGGTGTCACTAATCGTTACTCTGTCATGGAGTGGGGAGATGAGAACGGATGGCTGATGTATGTTCAACGTAGAGAGTGGACAACAGCGATAGGCGGTAACATCCAGTTAGTAGTAAACGGACAGATCATCACCCAAGGTGGAGCCATGACCGGTCAGCTAAAATTGCAGAATGGGCATGTTCTTCAATTAGAGTCCGCATCCGACAAGGCGCACTATATtctatc                               <  1:384847/301‑1 (MQ=255)`  
                                                                                                                                                                                                                                                                                     `ttCACGCTAACGGGCGCCTTTACCTGACCACAAACGGTTTTGACTGTGGCCAGTATCAACAGTTCTTTGGTGGTGTCACTAATCGTTACTCTGTCATGGAGTGGGGAGATGAGAACGGATGGCTGATGTATGTTCAACGTAGAGAGTGGACAACAGCGATAGGCGGTAACATCCAGTTAGTAGTAAACGGACAGATCATCACCCAAGGTGGAGCCATTACCGGTCAGCTAAAATTGCAGAATGGGCATGTTCTTCAATTAGAGTCCGCATCCGACAAGGCGCACTATATTCTATCTAAAGa                         <  1:610753/301‑1 (MQ=255)`  
                                                                                                                                                                                                                                                                                            `tataagagacagttACATGACCACAAACGGTTTTGACTGTGGCCAGTATCAACAGTTCTTTGGTGGTGTCACTAATCGTTACTCTGTCATGGAGTGGGGAGATGAGAACGGATGGCTGATGTATGTTCAACGTAGAGAGTGGACAACAGCGATAGGCGGTAACATCCAGTTAGTAGTAAACGGACAGATCATCACCCAAGGTGGAGCCATGACCGGTCGGCTAAAATTGCAGAATGGGCATGTTCTTCAATTAGAGTCCGCATCCGACAAGGCGCACTATATTCTATCTAAAGATGGTAAc                  <  1:324249/289‑1 (MQ=255)`  
                                                                                                                                                                                                                                                                                             `aaCGGGCGCCTTTACATGACCACAAACGGTTTTGACTGTGGCCAGTATCAACAGTTCTTTGGTGGTGTCACTAATCGTTACTCTGTCATGGAGTGGGGAGATGAGAACGGATGGCTGATGTATGTTCAACGTAGAGAGTGGACAACAGCGATAGGCGGTAACATCCAGTTAGTAGTAAACGGACAGATCATCACCCAAGGTGGAGCCATGACCGGTCAGCTAAAATTGCAGAATGGGCATGTTCTTCAATTAGAGTCCGCATCCGACAAGGCGCACTATATtctactgtctcttatacaca                 >  1:29397/1‑285 (MQ=255)`  
                                                                                                                                                                                                                                                                                             `aaCGGGCGCCTTTACATGACCACAAACGGTTTTGACTGTGGCCAGTATCAACAGTTCTTTGGTGGTGTCACTAATCGTTACTCTGTCATGGAGTGGGGAGATGAGAACGGATGGCTGATGTATGTTCAACGTAGAGAGTGGACAACAGCGATAGGCGGTAACATCCAGTTAGTAGTAAACGGACAGATCATCACCCAAGGTGGAGCCATGACCGGTCAGCTAAAATTGCAGAATGGGCATGTTCTTCAATTAGAGTCCGCATCCGACAAGGCGCACTATATTCTATCTAAAGactgtctct                 >  1:354378/1‑293 (MQ=255)`  
                                                                                                                                                                                                                                                                                               `aagagacagttACCTGACCACAAACGGTTTTGACTGTGGCCAGTATCAACAGTTCTTTGGTGGTGTCACTAATCGTTACTCTGTCATGGAGTGGGGAGATGAGAACGGATGGCTGATGTATGTTCAACGTAGAGAGTGGACAACAGCGATAGGCGGTAACATCCAGTTAGTAGTAAACGGACAGATCATCACCCAAGGTGGAGCCATGACCGGTCAGCTAAAATTGCAGAATGGGCATGTTCTTCAATTAGAGTCCGTATCCGACAAGGCGCACTATATTCTATCTAAAGATGGTAACAgg               <  1:504487/292‑1 (MQ=255)`  
                                                                                                                                                                                                                                                                                                 `gaggcagttACCTGAACAAAAACGGTTTTGACTGGGGCCAGTATCAAAAGTTCTTTGGTTGTTTTACTAATCGTTAAGTTGTTATGGGGTGGGGAGATGAGAACGGATGGCTGATGTATGTTCAACGTAGAGAGTGGACAACAGCGATAGGCGGTAACATCCAGTTAGTAGTAAACGGACAGATCATCACCCAAGGTGGAGCCATGACCGGTCAGCTAAAATTGCAGAATGGGCATGTTCTTCAATTAGAGTCCGCATCCGACAAGGCGCACTATATTCTATCTAAAGATGGTAACAGGaa             <  1:368445/294‑1 (MQ=255)`  
                                                                                                                                                                                                                                                                                                        `ttACCTGACCACAAACGGTTTTGACTGTGGCCAGTATCAACAGTTCTTTGGTGGTGTCACTAATCGTTACTCTGTCATGGAGTGGGGAGATGAGAACGGATGGCTGATGTATGTTCAACGTAGAGAGTGGACAACAGCGATAGGCGGTAACATCCAGTTAGTAGTAAACGGACAGATCATCACCCAAGGTGGAGCCATGACCGGTCAGCTAAAATTGCAGAATGGGCATGTTCTTCAATTAGAGTCCGCATCCGACAAGGCGCACTATATTCTATCTAAAGATGGTAACAGGAATAACTgg      >  1:330444/1‑301 (MQ=255)`  
                                                                                                                                                                                                                                                                                                        `ttACCTGACCACAAACGGTTTTGACTGTGGCCAGTATCAACAGTTCTTTGGTGGTGTCACTAATCGTTACTCTGTCATGGAGTGGGGAGATGAGAACGGATGGCTGATGTATGTTCAACGTAGAGAGTGGACAACAGCGATAGGCGGTAACATCCAGTTAGTAGTAAACGGACAGATCATCACCCAAGGTGGAGCCATGACCGGTCAGCTAAAATTGCAGAATGGGCATGTTCTTCAATTAGAGTCCGCATCCGACAAGGCGCACTATATTCTATCTAAAGATGGTAACAGGAATAACTgg      >  1:601741/1‑301 (MQ=255)`  

                                                                                                                                                                                                                                                                                                            `|`                                                                                                                                                                                                                                                                                                              
`CAGTGGCGCGATGAGACCAAGGGTTTCCGAGACGAAGCCAAGCGGTTCAAGAATACGGCTGGTCAATACGCTACATCTGCTGGGAACTCTGCTTCCGCTGCGCATCAATCTGAGGTAAACGCTGAGAACTCTGCCACAGCATCCGCTAACTCTGCTCATTTGGCAGAACAGCAAGCAGACCGTGCGGAACGTGAGGCAGACAAGCTGGAAAATTACAATGGATTGGCTGGTGCAATTGATAAGGTAGATGGAACCAATGTGTACTGGAAAGGAAATATTCACGCTAACGGGCGCCTTTACATGACCACAAACGGTTTTGACTGTGGCCAGTATCAACAGTTCTTTGGTGGTGTCACTAATCGTTACTCTGTCATGGAGTGGGGAGATGAGAACGGATGGCTGATGTATGTTCAACGTAGAGAGTGGACAACAGCGATAGGCGGTAACATCCAGTTAGTAGTAAACGGACAGATCATCACCCAAGGTGGAGCCATGACCGGTCAGCTAAAATTGCAGAATGGGCATGTTCTTCAATTAGAGTCCGCATCCGACAAGGCGCACTATATTCTATCTAAAGATGGTAACAGGAATAACTGGTACA  >  NC_001604.1/35215‑35815`

  

Alignment Legend

Aligned base mismatch/match (shaded by quality score): `ATCG/ATCG` `< 3 ≤` `ATCG/ATCG` `< 15 ≤` `ATCG/ATCG` `< 28 ≤` `ATCG/ATCG` `< 37 ≤` `ATCG/ATCG` `< 38 ≤` `ATCG/ATCG`

Unaligned base: `atcg`    Masked matching base: `atcg`    Alignment gap: `‑`    Deleted base: `‑`
